# German multicenter biobank and registry for phenotype-specific genomic research in thoracic aortic disease

**DOI:** 10.1007/s00423-026-04356-0

**Published:** 2026-10-24

**Authors:** Philipp Erhart, Daniel Körfer, Florian Simon, Alexander Gombert, Kyriakos Oikonomou, Eliane von Klitzing, Alexander Oberhuber, Hagen Kerndl, Anja Hofmann, Christian P. Schaaf, Evelin Hund, Dittmar Böckler

**Affiliations:** 1 Department of Vascular and Endovascular Surgery, Heidelberg University Hospital, Im Neuenheimer Feld 420, 69120 Heidelberg, Germany; 2 Department of Vascular and Endovascular Surgery, University Hospital of Düsseldorf, Düsseldorf, Germany; 3 Department of Vascular Surgery, University Hospital RWTH Aachen, Aachen, Germany; 4 Department of Vascular and Endovascular Surgery, University Hospital Frankfurt, Goethe University Frankfurt, Frankfurt am Main, Germany; 5 Clinic of Vascular and Endovascular Surgery, Helios Klinikum Berlin-Buch, Medical School Berlin, Berlin, Germany; 6 Department of Vascular and Endovascular Surgery, University Hospital of Münster, Münster, Germany; 7 Department of Vascular Surgery, Medical Faculty, University of Augsburg, Augsburg, Germany; 8 Division of Vascular and Endovascular Surgery, Department for Visceral, Thoracic and Vascular Surgery, Medical Faculty Carl Gustav Carus and University Hospital, Technische Universität Dresden, Dresden, Germany; 9 Institute of Human Genetics, Heidelberg University Hospital, Heidelberg, Germany

**Keywords:** Thoracic aortic disease, Stanford type B aortic dissection, Biobank, National registry, Genomics, Genotype–phenotype

## Abstract

**Purpose:**

To establish a national multicentre biobank and registry for future genomic and genotype–phenotype studies of distinct thoracic aortic disease entities and to report the study design and current recruitment status.

**Methods:**

A prospective multicenter registry with integrated biobanking was established in May 2024 across eight German vascular and cardiovascular centres. Patients with Stanford type B aortic dissection (STBAD), thoracic aortic aneurysm (TAA), intramural hematoma (IMH), or penetrating aortic ulcer (PAU) were enrolled. Clinical characteristics, treatment, imaging parameters, and longitudinal outcomes were collected in a centralized pseudonymized database, and EDTA blood samples were stored at − 80 °C for future genomic analyses. An established retrospective Heidelberg cohort of 118 patients with isolated STBAD serves as a hypothesis-generating cohort, while the prospective multicenter cohort provides an independent population for subsequent validation and extension of genetic findings. No genomic analyses were performed for the present report.

**Results:**

As of May 2026, 274 patients had been included: 118 patients with STBAD from the hypothesis-generating Heidelberg cohort and 156 patients from the prospective multicenter cohort comprising 84 patients with STBAD, 40 with TAA, 13 with IMH, and 19 with PAU. The most prominent descriptive difference between disease entities was age distribution. Among patients with STBAD, 116/202 (57.4%) were younger than 60 years, compared with 6/40 (15.0%) with TAA, 2/13 (15.4%) with IMH, and 2/19 (10.5%) with PAU. A positive family history of thoracic aortic disease was reported in 14 patients (5.1%), and hypermobility or other connective tissue features were documented in 12 patients (4.4%), and two patients (0.7%) had a documented connective tissue disorder. Overall, 217 patients (79.2%) underwent surgical or endovascular treatment. Median imaging follow-up ranged from 1.5 months in IMH to 48 months in the retrospective Heidelberg cohort.

**Conclusion:**

This national multicenter biobank and registry provides a deeply phenotyped platform for future investigation of the genetic basis of distinct thoracic aortic disease entities. The observed difference in age distribution provide a rationale for phenotype-specific genomic investigation but should not be interpreted as evidence of differing genetic contributions per se. Continued recruitment and longitudinal follow-up will enable future future genomic and genotype-phenotype analyses.

**Supplementary Information:**

The online version contains supplementary material available at https://doi.org/10.1007/s00423-026-04356-0.

## Introduction

Thoracic aortic disease (TAD) comprises a heterogeneous spectrum of potentially life-threatening disorders, including thoracic aortic aneurysm (TAA), Stanford type B aortic dissection (TBAD), intramural haematoma (IMH), and penetrating aortic ulcer (PAU). These entities differ in clinical presentation, natural history, and underlying pathology, suggesting that distinct biological mechanisms may contribute to their development and progression [1, 2].

A proportion of TAD is attributable to established monogenic aortopathies. Pathogenic variants in genes including FBN1, ACTA2, MYH11, MYLK, LOX, TGFBR1, and TGFBR2 are associated with increased susceptibility to thoracic aortic disease [3–7]. Current guidelines recommend genetic evaluation particularly in patients with young age at presentation, syndromic features, or a positive family history [1, 2]. However, established monogenic causes account for only a minority of sporadic cases, and the genetic architecture of most TAD remains incompletely understood [3, 8].

Familial aggregation and genome-wide studies support a substantial genetic contribution to TAD, including both rare high-impact variants and common genetic variation [8–11]. Many genetic studies have considered thoracic aortic aneurysm and dissection together under the term “thoracic aortic aneurysm and dissection” (TAAD). This classification has enabled large-scale analyses, but it may not account for differences in their underlying aetiology, disease progression and treatment response [11, 12]. Dedicated cohorts integrating detailed clinical phenotyping, longitudinal imaging, treatment data, and biospecimens are therefore needed to investigate distinct thoracic aortic disease entities separately.

Current genetic evaluation of thoracic aortic disease primarily focuses on the identification of pathogenic or likely pathogenic variants in established heritable thoracic aortic disease genes, usually through multigene panels and, in selected cases, broader sequencing approaches such as whole-exome or whole-genome sequencing [2, 8, 9]. Recent genomic studies have expanded this view by identifying common genetic risk loci for thoracic aortic aneurysm and dissection and by suggesting an interplay between rare pathogenic variants and polygenic risk [10, 11].

Acquired genomic alterations may represent additional mechanisms influencing thoracic aortic disease. Clonal haematopoiesis of indeterminate potential (CHIP) is an established risk modifier of atherosclerotic cardiovascular disease [13, 14], and emerging evidence suggests an association between JAK2 V617F-associated clonal haematopoiesis and thoracic aortic aneurysm [15, 16]. Other mechanisms, including somatic mosaicism, copy-number alterations, and structural variants, may not be adequately captured by conventional targeted genetic testing. Their potential contribution to phenotype-specific disease susceptibility and progression remains insufficiently characterized.

The aim of this study is to describe the design, structure, baseline cohort characteristics, and research objectives of a German multicenter biobank and registry for distinct thoracic aortic disease entities. The platform combines a retrospective, hypothesis-generating Heidelberg cohort of patients with isolated STBAD with an ongoing prospective multicenter cohort across German vascular and cardiovascular centres. Its primary purpose is to provide standardized phenotyping, longitudinal imaging, treatment data, and biospecimens for future genomic research.

## Materials and methods

### Study design

A national prospective multicenter observational registry with integrated biobanking was established in May 2024 to investigate patients with thoracic aortic disease in Germany. The present baseline analysis includes patients recruited or available for analysis between May 2024 and May 2026. The registry includes patients treated at eight vascular and cardiovascular centres with expertise in the management of thoracic aortic disorders. Patient recruitment was not restricted to the acute or subacute phase but also included patients during routine imaging surveillance or after previous surgical or endovascular treatment.

All participating centres obtained approval from their respective institutional ethics committees prior to patient recruitment. The study was conducted in accordance with the Declaration of Helsinki and applicable national data protection regulations. Written informed consent was obtained from all enrolled patients prior to inclusion and biological sampling. No genetic diagnostic testing or clinical genetic evaluation is performed as part of the study; generated genetic data are intended exclusively for scientific research. The registry is registered at German Clinical Trials Register (DRKS): DRKS00033646. The study protocol was approved by the institutional ethics committee of Heidelberg (S332-2023).

### Cohort design

**Fig. 1:**
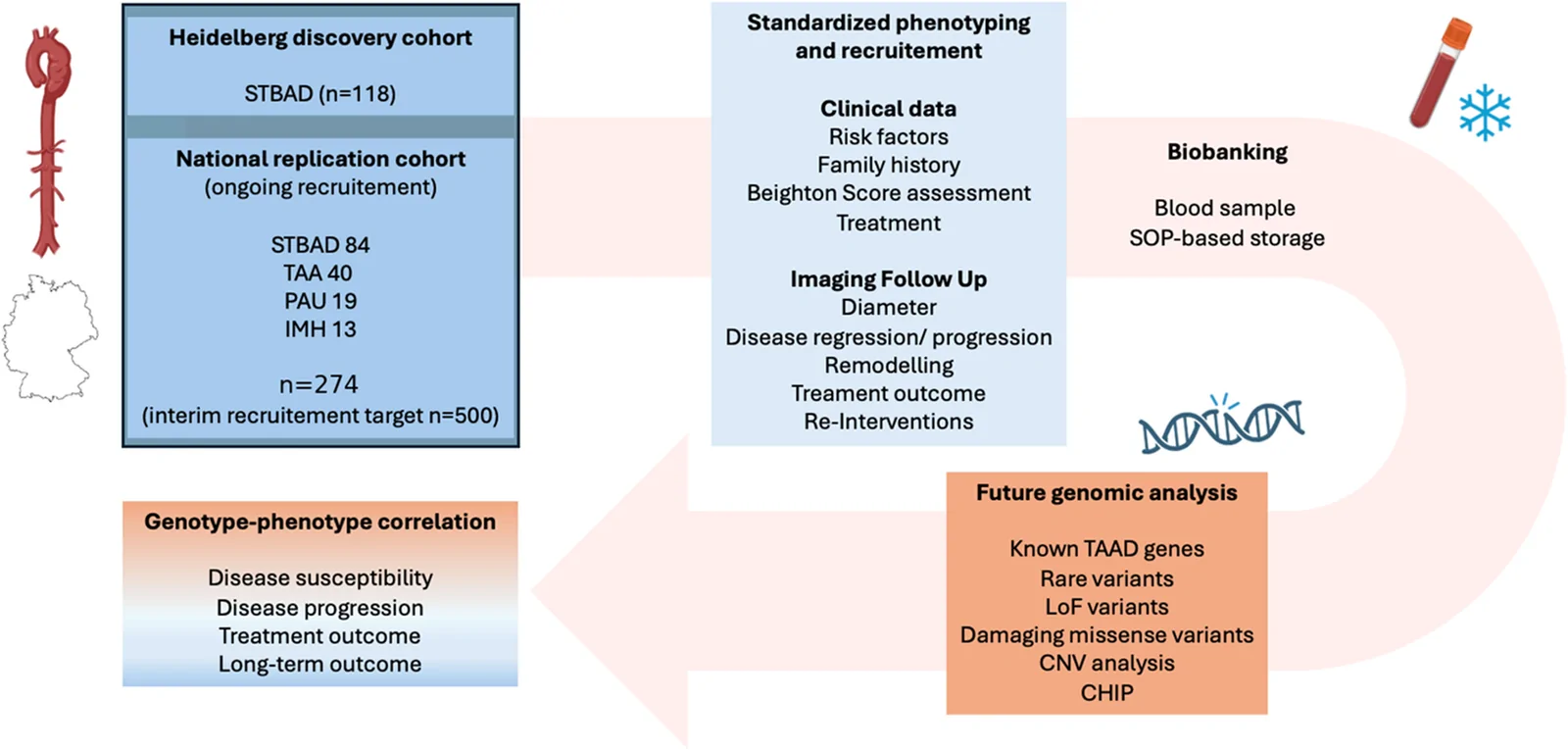
National Prospective Genomic Biobank and Registry for Thoracic Aortic Disease in Germany. The registry includes distinct TAD phenotypes and integrates clinical, imaging, treatment, and biospecimen data for future genomic and genotype–phenotype analyses. CHIP, clonal haematopoiesis of indeterminate potential; CNV, copy number variation; IMH, intramural haematoma; LoF, loss-of-function; PAU, penetrating aortic ulcer; STBAD, Stanford type B aortic dissection; TAAD, thoracic aortic aneurysm and dissection; TAA, thoracic aortic aneurysm. Generated with BioRender Online (SaaS)

The study comprises two complementary cohorts. An established retrospective Heidelberg biobank including 118 patients with isolated Stanford type B aortic dissection (STBAD) serves as a hypothesis-generating cohort. In parallel, a prospective multicenter cohort is being established across eight German centres and includes STBAD, thoracic aortic aneurysm, intramural haematoma, and penetrating aortic ulcer. The overall registry structure and planned genomic workflow are summarized in Fig. 1. The registry focuses on pathologies primarily originating in the descending thoracic aorta, excluding diseases primarily involving the ascending aorta or aortic arch. This anatomical restriction reflects the initial clinical focus oft he participating centres and aims to reduce phenotypic heterogeneity for subsequent genomic investigations.

The prospective multicenter cohort provides an independent population for subsequent validation and extension of candidate genetic findings and for genotype–phenotype analyses. Large publicly available reference datasets may be used for variant-frequency and enrichment analyses, while ancestry-matched external cohorts may be required for specific genetic association analyses.

### Data collection and follow-up

Clinical data are prospectively collected using standardized electronic case report forms. Variables include demographics, cardiovascular risk factors, comorbidities, clinical presentation, disease phenotype, family history, connective tissue features, treatment strategy, and longitudinal outcomes. Connective tissue features, including joint hypermobility, were assessed with reference to the revised Ghent criteria. CTA imaging data are collected at diagnosis and during follow-up, including aortic dimensions, disease extent and morphology, false lumen characteristics, treatment-related findings, aortic remodelling, progression, and reinterventions. Clinical imaging parameters are recorded in the central study database, whereas original imaging datasets remain stored locally at the participating centres for future applications. All clinical data are pseudonymized.

### Blood sampling and biobanking

Peripheral EDTA blood samples are collected according to standardized operating procedures and stored at − 80 °C in local biobank facilities. Following dedicated funding acquisition, planned genomic analyses will include established aortopathy genes, rare germline variants, structural variation, and acquired genomic alterations including clonal haematopoiesis of indeterminate potential (CHIP). Detailed sequencing, bioinformatic, and variant-prioritization strategies will be defined prospectively for individual genomic analyses.

### Sample size considerations

The retrospective Heidelberg cohort of 118 patients is intended primarily for hypothesis generation and exploratory assessment of established aortopathy genes and recurrent rare variants. The prospective multicenter cohort will provide an independent population for validation and extension analyses. A target cohort size of approximately 500 patients is considered a realistic recruitment milestone for exploratory analyses of recurrent rare variants and established aortopathy genes; variants below approximately 0.1% allele frequency will, however, be expected to occur only sporadically. The target therefore does not represent a definitive sample size for novel gene discovery or rare-variant association analyses, which will likely require continued recruitment and collaboration with additional cohorts.

Detailed inclusion and exclusion criteria are provided in Online Resource 1.

## Results

### Cohort characteristics

A total of 274 patients were included. The retrospective Heidelberg cohort consisted of 118 patients with isolated STBAD. The prospective multicenter cohort included 156 patients comprising 84 with STBAD, 40 with TAA, 13 with IMH, and 19 with PAU (Table 1). Within the prospective multicentre cohort, STBAD accounted for 53.8% and TAA for 25.6% of the total cohort. The most prominent descriptive difference between disease entities was age distribution. Among 202 patients with STBAD, 116 (57.4%) were younger than 60 years at disease onset, compared with 6/40 (15.0%) with TAA, 2/13 (15.4%) with IMH, and 2/19 (10.5%) with PAU (Table 2). The retrospective Heidelberg STBAD cohort had a lower median age at disease onset than the prospective multicentre cohort (56 vs. 68 years).

**Table 1:** Study cohort characteristics

Characteristic	STBAD Exploration (*n* = 118)	STBAD (*n* = 84)	TAA (*n* = 40)	PAU (*n* = 19)	IMH (*n* = 13)	Total (*n* = 274)
Male	82 (69.5%)	63 (75.0%)	18 (45.0%)	12 (63.2%)	8 (61.5%)	183 (66.8%)
Female	36 (30.5%)	21 (25.0%)	22 (55.0%)	7 (36.8%)	5 (38.5%)	91 (33.2%)
Age at disease onset (years)	56	68	70	71	69	—
Arterial hypertension	95 (80.5%)	72 (85.7%)	35 (87.5%)	18 (94.7%)	13 (100.0%)	233 (85.0%)
Diabetes	13 (11.0%)	7 (8.3%)	5 (12.5%)	6 (31.6%)	2 (15.4%)	33 (12.0%)
Hypercholesterinemia	20 (16.9%)	27 (32.1%)	20 (50.0%)	11 (57.9%)	2 (15.4%)	80 (29.2%)
Coronary heart disease	26 (22.0%)	8 (9.5%)	15 (37.5%)	7 (36.8%)	2 (15.4%)	58 (21.2%)
Smoking history	33 (28.0%)	34 (40.5%)	21 (52.5%)	8 (42.1%)	4 (30.8%)	100 (36.5%)
Drug consumption	4 (3.4%)	1 (1.2%)	0	0	0	5 (1.8%)
Familial history for TAAD	2 (1.7%)	11 (13.1%)	0	1 (5.3%)	0	14 (5.1%)
Hypermobility symptoms	5 (4.2%)	6 (7.1%)	0	0	1 (7.7%)	12 (4.4%)
Conservative treatment	29 (24.6%)	16 (19.0%)	3 (7.5%)	2 (10.5%)	6 (46.2%)	57 (20.8%)
Surgical treatment (endo/OR)	89 (75.4%)	68 (81.0%)	36 (90.0%)	17 (89.5%)	7 (53.8%)	217 (79.2%)
Imaging follow-up (median)	48 months	9 months	7 months	2 months	1.5 months	—
Further aneurysms or dissections	8 (6.8%)	13 (15.5%)	4 (10.0%)	3 (15.8%)	6 (46.2%)	34 (12.4%)
Connective tissue disorder	1 (0.8%)	1 (1.2%)	0	0	0	2 (0.7%)
Death	24 (20.3%)	4 (4.8%)	3 (7.5%)	0	0	31 (11.3%)

*IMH* intramural hematoma, *OR* surgical open repair, *PAU* penetrating aortic ulcer, *TAA* thoracic aortic aneurysm, *TAAD* thoracic aortic aneurysm and dissection, *STBAD* Stanford type B aortic dissection

**Table 2:** Distribution of younger patients < 60 years at disease onset

Thoracic aortic pathology	*n*	< 60 years	% <60
STBAD	202	116	57.4%
TAA	40	6	15.0%
IMH	13	2	15.4%
PAU	19	2	10.5%

*IMH* intramural hematoma, *PAU* penetrating aortic ulcer, *TAA* thoracic aortic aneurysm, *TAAD* thoracic aortic aneurysm and dissection, *STBAD* Stanford type B aortic dissection

A positive family history of thoracic aortic disease was present in 14 patients (5.1%). Hypermobility or other connective tissue features were documented in 12 patients (4.4%), and two patients (0.7%) had a documented connective tissue disorder. Further clinical characteristics and cardiovascular risk factors are summarized in Table 1.

### Treatment and longitudinal follow-up

Overall, 217 patients (79.2%) underwent surgical or endovascular treatment, while 57 (20.8%) were managed conservatively (Table 1). Median imaging follow-up was 48 months in the retrospective Heidelberg cohort, compared with 9 months for multicentre STBAD, 7 months for TAA, 1.5 months for IMH, and 2 months for PAU. The longer follow-up of the retrospective Heidelberg STBAD cohort should be considered when interpreting cumulative event frequencies. Further aneurysmatic disease or arterial dissections were documented in 34 patients (12.4%), and 31 patients (11.3%) died during available follow-up (Table 1).

At the time of analysis, biological samples from 274 patients were available; genomic sequencing had not yet been initiated.

## Discussion

This study establishes a national multicentre biobank and registry for distinct thoracic aortic disease entities in Germany, integrating clinical phenotyping, longitudinal imaging, treatment data, and biospecimen collection. The current cohort comprises 274 patients, including 156 prospectively recruited patients across eight centres and a retrospective, hypothesis-generating Heidelberg cohort of 118 patients with STBAD. Genomic sequencing has not yet been initiated.

The most prominent descriptive difference between disease entities was the age distribution. More than half of patients with STBAD (57.4%) presented before the age of 60, compared with approximately 10–15% of patients with TAA, IMH, or PAU. This finding is consistent with observations from large registries such as the International Registry of Acute Aortic Dissection (IRAD), in which STBAD generally affects a younger patient population than degenerative aortic pathologies [17, 18]. Current guidelines support genetic evaluation in patients with thoracic aortic disease presenting at a young age, particularly in the presence of a family history or syndromic features [1, 2]. Systematic assessment of family history and connective tissue features within the registry may help identify patients who might otherwise not undergo detailed clinical genetic assessment in routine practice. Importantly, younger age at presentation in STBAD should not be interpreted as evidence of a stronger genetic contribution per se, but provides a rationale for phenotype-specific genomic investigation.

Clinical genetic diagnostics in vascular medicine require structured phenotypic assessment, family history, and appropriate genetic counselling, with testing strategies tailored to the suspected vascular phenotype [19, 20]. The present registry deliberately separates this clinical diagnostic pathway from research sequencing: genetic analyses within the registry are intended exclusively for scientific purposes and do not constitute clinical genetic testing.

The marked age difference supports the rationale for investigating aortic dissection and aneurysmal disease as separate phenotypes rather than combining them under the conventional TAAD classification. Large-scale genomic studies have demonstrated a complex genetic architecture of TAAD involving both rare and common genetic variation [9–11]. Although genetic predispositions may affect multiple aortic segments, many existing genetic studies combine thoracic aortic aneurysms and dissection under the broad classification of TAAD. The present registry therefore emphasizes both anatomical localization and distinct disease phenotypes to facilitate more specific genotype-phenotype investigations. The comparisons in the present study are descriptive, however, and should not be interpreted as population-based estimates of phenotype differences, because referral patterns, cardiovascular risk profiles, treatment selection, and cohort composition may differ between disease groups.

A major strength of the registry is the combination of phenotype-specific classification, longitudinal imaging, and biospecimen collection. The substantial proportion of patients undergoing surgical or endovascular treatment, provide an opportunity to investigate treatment-related outcomes in addition to disease susceptibility. Genotype–phenotype analyses may subsequently be extended to outcomes after conservative treatment or TEVAR, including aortic progression, reintervention, and stent-graft-induced new entry (SINE). The longitudinal imaging data may further allow genotype-based comparisons of aortic remodelling, treatment response or complications.

The registry also offers opportunities for future genotype–radiology analyses. Centralized imaging parameters can be combined with locally stored imaging datasets, and future analyses may incorporate semi-automated or automated image analysis to quantify aortic morphology, remodelling, and disease progression.

The discovery–validation structure represents another important feature. The retrospective Heidelberg cohort provides a hypothesis-generating population, while the prospective multicentre cohort offers an independent population for subsequent replication and validation. Initial sequencing analyses are planned for the discovery cohort and may be used to prioritize candidate variants and genes, followed by targeted evaluation in the prospective cohort, and, where required, external ancestry-matched datasets. The planned expansion toward approximately 500 patients should be regarded as a recruitment milestone rather than a definitive sample size for novel gene discovery.

Acquired genomic alterations represent an additional exploratory research direction. CHIP is an established risk modifier of atherosclerotic cardiovascular disease [13, 14], while its specific role in thoracic aortic disease remains poorly defined. Emerging observations linking JAK2 V617F-associated clonal haematopoiesis to thoracic aortic aneurysm support further investigation [15, 16]. The available blood samples may also permit exploratory assessment of somatic mosaicism, copy-number changes, and other structural variants, and their potential associations with disease phenotype and progression.

The study has several limitations. The combination of retrospective and prospective cohorts results in differences in data completeness and follow-up duration. In particular, the longer follow-up of the retrospective Heidelberg cohort increases the opportunity to capture mortality and subsequent aortic events. Therefore, crude event frequencies should not be directly compared between cohorts without accounting for observation time. The high proportion of treated patients may reflect referral and selection patterns of specialized vascular centres, with uncomplicated patients managed outside specialized centres potentially being underrepresented. Furthermore, recruitment included patients at different disease stages, including after previous treatment or during routine imaging surveillance. Therefore, intervention rates are not directly comparable with those reported in acute dissection registries such as IRAD [18]. Accordingly, observed differences between disease entities are descriptive and should not be interpreted as population-based differences. Original imaging datasets remain at the participating centres, which currently limits centralized image re-analysis and requires harmonization of imaging parameters across sites. Finally, the relatively small IMH and PAU subgroups currently limit phenotype-specific analyses.

Subject to funding, sequencing capacity, and completion of sample processing, the registry is expected to support initial exploratory genomic analyses during continued recruitment. Continued recruitment and standardized longitudinal follow-up will strengthen the registry and allow future assessment of genetic determinants, treatment response, and disease progression across distinct thoracic aortic phenotypes.

We invite additional German and international centres with an interest in thoracic aortic disease and genomic research to participate in the registry; interested centres may contact the corresponding author directly.

## Conclusion

This German multicentre biobank and registry provides a standardized platform integrating clinical phenotyping, longitudinal imaging, treatment data, and biospecimen collection for distinct thoracic aortic disease entities. The observed differences in age distribution between STBAD and other thoracic aortic phenotypes provide a rationale for their separate investigation in future genomic studies. The high proportion of treated patients and longitudinal imaging data additionally provides a basis for investigating genotype–treatment and genotype–radiology associations, including aortic remodelling and long-term outcomes after different treatment modalities. Continued recruitment, longer standardized follow-up, and future genomic sequencing will be required to determine the registry’s value for robust genotype–phenotype analyses and for evaluating disease progression and treatment outcomes across distinct thoracic aortic phenotypes.

## Supplementary Information

Below is the link to the electronic supplementary material.

Supplementary Material 1

## Data Availability

The data underlying this report are held within the participating centres and the central study infrastructure; availability for secondary analyses is subject to ethics approval, data-protection requirements, and applicable study governance.
