# Supplementary material for: German multicenter biobank and registry for phenotype-specific genomic research in thoracic aortic disease
Source: Langenbecks Arch Surg. 2026 Oct 24;411(1):292. doi: 10.1007/s00423-026-04356-0 (PMC13710571; doi:10.1007/s00423-026-04356-0)
Supplement: Supplementary file 1 [file 423_2026_4356_MOESM1_ESM.docx]

# Supplementary Information

German Multicenter Biobank and Registry for Phenotype-Specific Genomic Research in Thoracic Aortic Disease

Journal: Langenbeck’s Archives of Surgery

Authors: Philipp Erhart, Daniel Körfer, Florian Simon, Alexander Gombert, Kyriakos Oikonomou, Eliane von Klitzing, Alexander Oberhuber, Hagen Kerndl, Anja Hofmann, Christian Schaaf, Evelin Hund, Dittmar Böckler

Corresponding author: Philipp Erhart, Department of Vascular and Endovascular Surgery, Heidelberg University Hospital, Im Neuenheimer Feld 420, 69120 Heidelberg, Germany; [philipp.erhart@med.uni-heidelberg.de](mailto:philipp.erhart@med.uni-heidelberg.de)

**Online Resource 1. Supplementary Table 1. Inclusion and Exclusion Criteria of the Study Register**

|  | **Criteria** |
| --- | --- |
| **Inclusion criteria** |  |
| Age | Adult patients ≥18 years |
| Consent | Written informed consent and ability to provide informed consent |
| Diagnosis | Confirmed diagnosis of thoracic aortic pathology, including penetrating aortic ulcer (PAU), intramural haematoma (IMH), Stanford type B aortic dissection (STBAD), or thoracic aortic aneurysm |
| Anatomical location | Primary origin of the aortic pathology within the thoracic aorta, without involvement of the aortic arch or ascending aorta as the primary disease location |
| Disease extension | Extension into the abdominal aorta or iliac arteries is permitted for Stanford type B aortic dissection |
|  |  |
| Exclusion criteria |  |
| Consent | Missing informed consent |
| Age | Age <18 years |
| Data protection | Insufficient pseudonymization/anonymization of patient data |
| Aortic pathology | Traumatic aortic diseases (e.g., following accidents or external trauma), thoracoabdominal pathologies (e.g. thoracoabdominal aortic aneurysms Crawford type II, III, IV, V) |
| Data transfer | Data transfer outside the established Heidelberg Medical Data Model platform (https://medical-data-models.org) |
| Genetic testing | No exclusion based on genetic findings; genetic analyses are performed exclusively for scientific purposes and do not represent clinical genetic diagnostics |
